# Laryngeal Cancer in the Modern Era: Evolving Trends in Diagnosis, Treatment, and Survival Outcomes

**DOI:** 10.3390/jcm14103367

**Published:** 2025-05-12

**Authors:** Alexandru-Romulus Hut, Eugen Radu Boia, Diana Para, Gheorghe Iovanescu, Delia Horhat, Loredan Mikša, Maria Chiriac, Raphaël Galant, Alexandru Catalin Motofelea, Nicolae Constantin Balica

**Affiliations:** 1 Department of Doctoral Studies, “Victor Babes” University of Medicine and Pharmacy Timisoara, Eftimie Murgu Square No. 2, 300041 Timisoara, Romania; alexandru.hut@umft.ro (A.-R.H.); diana.para@umft.ro (D.P.); alexandru.motofelea@umft.ro (A.C.M.); 2 ENT Department, “Victor Babes” University of Medicine and Pharmacy Timisoara, Eftimie Murgu Square No. 2, 300041 Timisoara, Romania; giovanescu@umft.ro (G.I.); horhat.ioana@umft.ro (D.H.); miksa.loredan@yahoo.com (L.M.); maria.boleachiriac@gmail.com (M.C.); balica@umft.ro (N.C.B.); 3 ENT Department, Emergency City Hospital, 300254 Timisoara, Romania; 4 Hôpital Européen Georges-Pompidou, Assistance Publique-Hôpitaux de Paris, Université Paris Cité, 20 Rue Leblanc, 75015 Paris, France; raphaelumf@gmail.com; 5 Center for Molecular Research in Nephrology and Vascular Disease, Faculty of Medicine, “Victor Babes” University of Medicine and Pharmacy, 300041 Timisoara, Romania

**Keywords:** laryngeal cancer, squamous cell carcinoma, epidemiology, risk factors, biomarkers, treatment

## Abstract

**Background/Objectives**: Laryngeal cancer (LC), predominantly squamous cell carcinoma (SCC), represents a considerable health burden worldwide. Tumour subsite heterogeneity (supraglottic, glottic, subglottic) influences clinical behavior and outcomes. This review synthesizes current knowledge on epidemiology, risk factors, diagnostics, histological variants, biomarkers, treatment modalities, and survival. **Results**: This narrative review synthesizes current literature on the epidemiology, risk factors, diagnosis, histological variants, biomarkers, and prognosis of LC. The review highlights the critical influence of tumour sites (supraglottic, glottic, subglottic) on metastatic patterns and survival. Key risk factors of LC include tobacco and alcohol use, human papillomavirus (HPV) infection, and occupational exposures. The diagnostic process encompasses clinical examination, endoscopy, biopsy, and imaging. Several biomarkers that aid in diagnosis, treatment plan determination, and prognosis prediction have been established. These biomarkers include long noncoding RNAs, cell cycle regulators, apoptosis regulators, oncogenes, tumour suppressor genes, growth factor pathway components, angiogenic factors, structural proteins, sex hormone receptors, and immunological markers. Current treatment modalities range from organ-preserving surgery and radiotherapy to combined chemoradiotherapy and total laryngectomy. Finally, survival data are presented and stratified by stage and subsite. **Conclusions**: The review underscores the need for a multidisciplinary approach to LC management, integrating clinical, pathological, and molecular information to optimize patient outcomes.

## 1. Introduction

Cancers of the head and neck collectively represent the seventh most common type of cancer worldwide, encompassing a diverse array of tumours that originate within the upper aerodigestive tract [1]. According to global cancer statistics for 2022, laryngeal cancer (LC) is among the most prevalent cancers, with 188,960 new cases and 103,216 deaths reported [2]. The vast majority of these—between 85% and 95%—are classified as squamous cell carcinomas (SCC) [3]. The epidemiology of LC reveals a substantial burden. In 2022, global incidence rates reached over 165,598 cases in men and over 23,362 in women, with associated deaths exceeding 90,256 and 12,960, respectively [2].

Geographically, Cuba (7.8 per 100,000) and Montenegro (7.0 per 100,000) report the highest incidence rates, while Eswatini (0.18 per 100,000) and Cameroon (0.31 per 100,000) have the lowest. Asia is the most prevalent continent for LC, followed by Europe, whilst the epidemiologic burden in Africa remains low [4]. Within Europe, a high-prevalence region, incidence varies considerably; Spain reports rates above 12 per 100,000, while the UK’s rates are below 5 per 100,000 [5]. In Romania, oral malignant tumors are prevalent, with approximately 2388 new cases annually [6].

Regarding LC mortality, Cuba (3.9 per 100,000) and Montenegro (3.5 per 100,000) also register high mortality rates, in contrast to Iceland (0.08 per 100,000) and Martinique (0.11 per 100,000), which have the lowest [4]. These variations are linked to socioeconomic factors, with higher incidence in areas with lower average incomes and education [7]. Regarding gender differences, in 2021, the age-standardized DALY rate of LC in males was approximately 7.13 times higher than that in females, with males experiencing 282.12 cases per 100,000 individuals, compared to 39.59 cases per 100,000 individuals in females [8]. Higher Human Development Index (HDI) regions tend to have higher incidence but lower mortality, reflecting disparities in healthcare access and quality, while lower Sociodemographic Index (SDI) regions experience higher mortality rates [9,10]. Key etiological factors include tobacco smoking [11,12], alcohol consumption [13,14,15], high-risk human papillomavirus (HPV) infection [16], and occupational exposures such as asbestos [17,18].

Characteristic clinical manifestations often prompt the diagnosis of LC. These symptoms, which can vary significantly depending on the tumour’s location and size, commonly include a persistent lump or non-healing sore, ongoing throat pain, difficulty swallowing (dysphagia), and alterations in voice quality, such as hoarseness [19]. Despite significant advancements in instruments like flexible laryngoscopes, surgical techniques, and chemoradiation therapy, the mortality rate remains high; the 5-year survival rate is 64% because approximately two-thirds of patients are diagnosed with advanced cancer, leading to a poor prognosis [20]. Furthermore, even with improvements in treatment modalities, the American Cancer Society has reported a tendency for the 5-year survival rate for laryngeal cancer patients to decline [21].

Treatment options for LC include surgery, radiation therapy, and chemotherapy, used alone or in combination [22,23]. Recent advancements in immunotherapy and targeted therapy have significantly impacted laryngeal cancer treatment, offering hope for patients with recurrent or advanced disease. These therapies improve progression-free and overall survival rates, particularly in combination. The integration of immunotherapy with targeted therapies like anti-EGFR has shown promising results in tumor response, and is increasingly considered for managing recurrent locoregionally advanced squamous cell carcinoma of the head and neck, including laryngeal cancer [24]. A complex interplay of factors determines a patient’s prognosis in LC, broadly categorized as relating to the host, the tumour itself, or the treatment strategy [25]. Host factors encompass individual characteristics such as age, sex, nutritional status, general health and physical condition, coexisting medical conditions’ presence, and the immune response’s robustness. Tumour-related factors include the primary location of cancer, its TNM stage (which reflects the size, involvement of lymph nodes, and presence of distant metastasis), the microscopic grade (degree of abnormality), and whether any other primary cancers are present concurrently [25]. While current staging systems predominantly focus on tumour-related characteristics, a complete and accurate assessment of prognosis requires consideration of host and tumour factors. In addition, correctly determining the treatment plan is essential for accurately estimating the prognosis [26].

This review explore recent advancements in molecular biomarkers, histopathological subtypes, and innovative therapeutic approaches particularly immuno- and targeted therapies to enhance prognostic stratification and inform treatment decisions in laryngeal cancer. Our objectives are to assess emerging biomarkers for personalized risk evaluation, delineate the clinical and molecular diversity of LC subtypes, and propose an integrated management framework that optimizes both survival and functional outcomes.

## 2. Materials and Methods

To identify relevant literature, a comprehensive search was conducted using electronic databases such as PubMed/MEDLINE, Scopus, and Web of Science. The search strategy combined both Medical Subject Headings (MeSH) and free-text terms related to “laryngeal cancer”, “laryngeal carcinoma”, and “squamous cell carcinoma”, along with additional terms addressing specific aspects of the review (for example, “epidemiology”, “risk factors”, “diagnosis”, “histological variants”, “biomarkers”, “treatment”, “survival”, and “quality of life”). Searches were performed to capture all publications available from the inception of each database until March 2025. In order to ensure a comprehensive review, reference lists from the identified articles, including relevant review articles and original studies, were also examined.

Articles were considered eligible if they addressed key aspects of laryngeal cancer, particularly those relating to squamous cell carcinoma—and if they included information on epidemiology, risk factors, diagnostic methods, treatment strategies, prognostic biomarkers, survival outcomes, or quality-of-life measures. Only studies published in peer-reviewed journals and written in English were included. The review focused on original research articles, systematic reviews, meta-analyses, and established clinical guidelines. Articles such as isolated case reports or very small case series, studies focused solely on other head and neck cancers without specific details regarding LC, or non-peer-reviewed materials, abstracts, or conference proceedings with limited methodological detail were excluded from this review.

Because this review takes a narrative approach, the extracted data were synthesized qualitatively rather than quantitatively. Major themes were identified and then organized into coherent subsections that correspond to the different facets of LC. This approach facilitated an integrated discussion of the complex interplay between epidemiological trends, clinical parameters, molecular insights, treatment options, and patient outcomes.

## 3. Results

### 3.1. Risk Factors of LC

**Figure 1:**
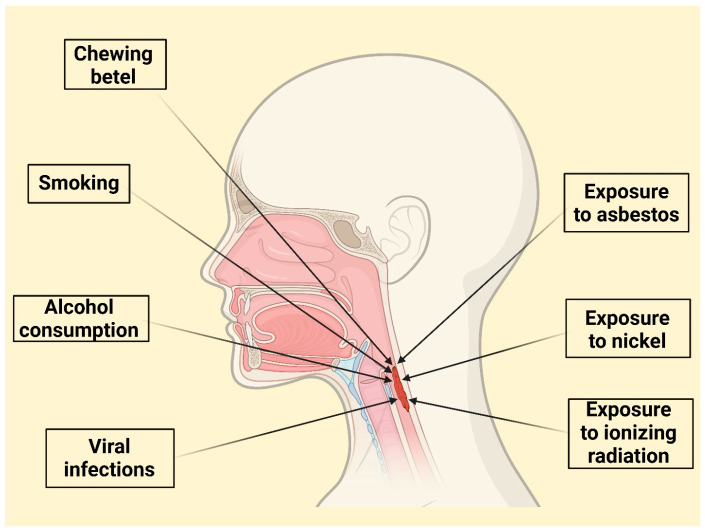
Overview of Key Lifestyle, Viral, and Environmental Risk Factors in Laryngeal Cancer Development.

A combination of lifestyle and environmental factors significantly influences LC development (Figure 1).

Undoubtedly, tobacco use is the most prominent risk factor [27]. The risk of LC increases substantially with both the duration and intensity of smoking, exhibiting a dose-response relationship. However, this relationship may not be perfectly linear, with a possible “saturation effect” at very high levels of consumption (more than 20 years and over 30 cigarettes per day) [11,12]. Smoking cessation reduces risk, but it remains elevated for up to 15 years after quitting [28]. The synergistic effect of tobacco’s carcinogens is amplified by human papillomavirus [16]. Cigarette smoking correlates with a sevenfold risk increase of LC [11]. Passive smoking also contributes to LC deaths [28]. Smokers have a higher likelihood of dying from LC [29]. Black smokers demonstrate a higher risk level than white smokers [30].

Excessive alcohol consumption is a significant independent risk factor for LC, exhibiting a clear dose–response relationship where both the amount and duration of intake proportionally elevate the risk [13,14,15]. Alcohol accounts for a substantial portion of laryngeal cancer-related mortality, especially in regions with high alcohol consumption levels [31]. In Europe, approximately 30% of individuals who died from LC were identified as alcoholics [31]. Moreover, the global contribution of alcohol to the incidence of LC may be on the rise [32].

While traditional risk factors like tobacco and alcohol remain significant, emerging concerns and socioeconomic disparities require further attention. Although initially marketed as a safer alternative, e-cigarettes have raised concerns due to the presence of potentially carcinogenic substances in some e-liquids [33]. A Korean study found formaldehyde and acetaldehyde in all 225 tested e-liquids [34]. Both of the compounds are classified as Group 1 carcinogens with links to head and neck cancers [35]. *N′*-nitrosonornicotine (NNN), a TSNA, has been shown to induce head and neck tumours in animal studies [36]. PAHs, like 1-hydroxypyrene (1-HOP) and benzopyrene, have demonstrated carcinogenic effects on the upper respiratory tract in animal models [36,37]. While in vivo studies on e-cigarettes and laryngeal mucosa are limited, one study in rats showed non-statistically significant hyperplasia and metaplasia after four weeks of exposure to e-cigarette aerosols [38].

Opium, classified as a human carcinogen by the IARC (International Agency for Research on Cancer), has shown preliminary links to increased head and neck squamous cell carcinoma (HNSCC) risk, including LC, although more research is needed to solidify this connection. The mechanism likely involves the carcinogenic alkaloids present in opium, which can induce DNA damage and mutations upon metabolic activation [39,40].

Beyond tobacco and alcohol, certain viral infections, notably high-risk strains of human papillomavirus (HPV), have been implicated in LC development [41].

Over the past few decades, HPV has been recognized as a key etiological agent, especially in oropharyngeal squamous cell carcinoma (OPSCC), where HPV-positive tumors form a distinct clinical and molecular subgroup compared with their HPV-negative counterparts [42]. However, the etiological role of HPV in LC appears to vary significantly by geographic region, with a lower prevalence in some areas, suggesting it is not always a primary cause [43,44,45]. Moreover, EBV has been suggested as a risk factor for LC. EBV’s genome and latent protein EBNA have been found in malignant laryngeal cells, suggesting a potential role as a risk factor or cofactor, though its presence in LSCC can be inconsistent [46]. EBV, like HPV, can produce oncoproteins that disrupt cell cycle control and promote uncontrolled cell growth, contributing to carcinogenesis [47].

Other identified risk factors include occupational exposure to asbestos [17,18] and possibly nickel or ionizing radiation [48]. Chewing betel, particularly in combination with tobacco and alcohol, substantially elevates risk, specifically in specific populations like Taiwan [49,50]. *Helicobacter pylori* infection has also been linked to an increased risk of LC [51], as has gastroesophageal reflux disease (GERD) and laryngopharyngeal reflux (LPR), likely due to chronic inflammation. Chronic inflammation leads to the release of reactive oxygen species (ROS) and inflammatory mediators, which can damage DNA, promote cell proliferation, and create a microenvironment conducive to tumour development [52,53,54]. Moreover, metabolic syndrome and its associated components (high blood glucose, increased waist circumference, elevated triglycerides, high blood pressure, and low HDL cholesterol) have also been identified as independent risk factors [55,56].

The oral and throat microbiome is emerging as a significant factor. Differences in bacterial composition, particularly elevated levels of genera like *Fusobacterium*, *Prevotella*, and *Streptococcus*, have been observed in LC patients compared to healthy individuals [57,58]. *Fusobacterium*, an invasive anaerobe, may contribute to chronic inflammation and carcinogenesis, and shifts in the oral microbiome could serve as an early marker of LC [58,59]. The microbiome’s influence extends to immune system modulation, metabolic regulation, and even cancer promotion, with interactions with alcohol and tobacco further contributing to LSCC development. Certain bacteria can produce carcinogenic metabolites, exacerbate inflammation, and suppress anti-tumor immune responses [60,61,62,63].

LC outcomes are significantly worsened by delays in diagnosis and treatment. Socioeconomic disparities such as lack of health insurance and limited healthcare access delaying cancer screening contribute to these delays and to the number of deaths from modifiable risk factors such as tobacco, alcohol and occupational exposures [64,65]. Men tend to have an increased risk of LC [66,67,68] and have a poor prognosis in comparison to women [69]. Preventive measures, particularly reducing tobacco and alcohol consumption and improving access to screening, are crucial for mitigating the burden of this disease [70,71]. Furthermore, in 2021, attributable deaths due to tobacco, occupational risks, and alcohol were 66.46%, 5.92%, and 12.4%, respectively. While deaths attributable to these factors have generally decreased since 1990, deaths due to occupational risks have increased in females. The proportion of LC deaths attributable to tobacco was highest in high-middle sociodemographic index (SDI) regions (76.15%), while high SDI regions had the highest proportions attributable to occupational risks (11.56%) and alcohol (19.31%) [72]. Country-specific data reveals that Armenia (middle SDI) had the highest proportion of LC deaths attributable to tobacco (83.86%), while the UK (high SDI) had the highest proportion linked to occupational risks (20.11%), and Czechia (high SDI) had the highest proportion attributable to alcohol (27.35%). These disparities highlight the complex interplay between individual behaviours, environmental exposures, and access to preventative care and treatment, underscoring the need for targeted interventions [72].

### 3.2. Histological Subtypes of LC

Laryngeal squamous cell carcinoma (LSCC), the most prevalent malignancy of the larynx, demonstrates a spectrum of differentiation, broadly categorized as keratinizing or non-keratinizing. Keratinizing SCC, typically encompassing well-differentiated and moderately differentiated forms, exhibits significant keratin production, often forming characteristic keratin pearls and demonstrating abundant intracellular keratin. In contrast, non-keratinizing SCC, usually associated with poorly differentiated tumours, lacks this well-developed keratinization; in these cases, immunohistochemical markers like CK5/6, p63, p40, and EMA are crucial to confirm the epithelial origin [73]. Beyond this broad distinction, laryngeal SCC encompasses several distinct variants, each with unique morphological features, clinical behaviours, and prognostic implications, as summarized in Table 1.

In contrast, papillary/exophytic SCC (PSCC/ESCC) demonstrates a predominantly exophytic or papillary growth pattern with frank cytomorphologic malignancy [74]. Verrucous carcinoma (VC) is a well-differentiated, slow-growing form characterized by a pushing border of infiltration, abundant keratinization, and a lack of cytologic atypia [75]. Spindle cell (sarcomatoid) SCC (SCSCC) is a biphasic tumour with both epithelial and spindle cell components, often presenting as a polypoid mass [76,77]. Basaloid SCC (BSCC) is a high-grade variant with a prominent basaloid component and often aggressive behaviour [78]. Adenosquamous carcinoma (ASC) is characterized by an admixture of SCC and adenocarcinoma components [79,80,81]. Distinguishing between keratinizing and non-keratinizing SCC and accurately classifying these variants is essential for guiding treatment decisions and predicting patient outcomes. Careful histological evaluation, often supplemented by immunohistochemistry, is paramount.

### 3.3. Diagnosis

Accurate diagnosis of LC relies on a thorough patient history, a comprehensive physical examination, and appropriate diagnostic procedures. The patient’s history should include detailed inquiries about risk factors like tobacco and alcohol use, current medications, and any coexisting medical conditions that might influence treatment decisions [82]. The clinical presentation of LC varies considerably depending on the tumour’s location and size. Glottic tumours often present early with hoarseness, while supraglottic tumours may manifest later with symptoms such as pain, persistent hoarseness, or dysphagia (difficulty swallowing) [82]. The diagnostic modalities of LC are summarized in Table 2. Direct visualization of the larynx is essential, typically achieved through indirect laryngoscopy, flexible fiberoptic laryngoscopy, or video stroboscopy. Videostroboscopy, in particular, demonstrates high sensitivity (96.8%) and specificity (92.8%) in predicting the invasiveness of laryngeal lesions [83]. Tissue confirmation through biopsy of the primary tumour or fine-needle aspiration of suspicious lymph nodes is crucial for definitive diagnosis. Imaging studies play a vital role in staging cancer. Computed tomography (CT) is valuable for assessing bone involvement, while positron emission tomography combined with CT (PET/CT) helps detect recurrences, local and nodal spread, and distant metastases [84]. Magnetic resonance imaging (MRI) offers superior sensitivity (80%) and specificity (92.9%) compared to CT (60% sensitivity, 85.7% specificity) in evaluating cartilage and soft tissue invasion [85]. Narrow-band imaging (NBI) has also emerged as a highly sensitive (97%) and specific (92.5%) technique for identifying both LC and its precursor lesions [86].

Traditional diagnostic approaches for LC, such as imaging and tissue biopsy, are fundamental but can be limited in detecting early-stage disease. Over the last decade, there has been increasing interest in using liquid biopsies to detect cancer-specific biomarkers in patients’ body fluids [87,88]. Liquid biopsy has been reported to play roles in early malignancy detection in diverse tumor types [89,90]. As a rapid and noninvasive approach, liquid biopsies have emerged as an exciting investigational avenue to obtain information on cancer diagnosis, treatment response, and progression [91,92]. Liquid biopsies are minimally invasive tests analyzing biomarkers in fluids like blood and saliva. These biomarkers, including circulating tumour cells (CTCs), circulating tumour DNA (ctDNA), exosomes carrying microRNAs (miRNAs), and even oral microbiome constituents, offer potential for earlier detection, refined prognosis, and real-time monitoring of treatment response in HNSCC, and specifically LC [93,94].

Since LC is frequently diagnosed at later stages, methods for earlier identification are critical. Research has demonstrated that ctDNA, fragments of DNA released by tumour cells, are detectable in the plasma and saliva of patients with LSCC, with levels correlating to the disease stage [95]. Epigenetic modifications, such as aberrant DNA methylation, also hold diagnostic promise. Studies have identified specific gene methylation patterns in plasma or serum associated with the early detection of respiratory cancers, including LSCC [96,97,98,99]. Furthermore, analysis of the oral microbiome, specifically detecting certain microbiota in mouthwash, has emerged as a novel liquid biopsy approach for LSCC [58].

Moreover, liquid biopsies provide valuable prognostic information for LC patients and enable monitoring of treatment response. Analyzing circulating tumour cells (CTCs), circulating tumour DNA (ctDNA), and microRNAs (miRNAs) is key. Higher preoperative CTC counts [100,101,102] and ctDNA hypermethylation [98] correlate with worse outcomes in LSCC. Changes in these markers and specific miRNAs [103,104,105,106] are linked to treatment and survival. Furthermore, liquid biopsies allow for minimally invasive, repeated sampling to dynamically monitor treatment efficacy and detect recurrence. Changes in CTC and ctDNA levels indicate treatment response or resistance [100,101,107].

Traditional diagnostic methods, relying on clinical examination, endoscopy, and histopathological analysis of biopsies, can be time-consuming and subject to inter-observer variability [108]. AI, particularly through machine learning (ML) and deep learning (DL) techniques, offers the potential to improve diagnostic accuracy and efficiency. In LC, various imaging modalities are used to train AI algorithms. Studies have utilized histological whole-slide images (WSI) [109] and endoscopic/clinical imaging [110]. For example, stimulated Raman scattering histology integrated with DL algorithms has shown an accuracy of 90% in diagnosing laryngeal SCC [111]. Other approaches have used traditional ML techniques to classify laryngeal tissues as normal or malignant based on textural information from narrow-band endoscopic images, achieving high recall rates [110]. These AI-driven diagnostic tools have the potential to assist clinicians in earlier and more accurate detection of LC, potentially leading to improved patient outcomes.

In addition, AI models, particularly deep neural networks (DNNs), can analyze diverse clinical data to provide more personalized risk assessments. A study by Choi et al., (2023) demonstrated that a DNN model incorporating various clinical factors significantly outperformed models using only the TNM stage for predicting survival in LSCC patients [111]. This aligns with previous findings highlighting the importance of factors like age, performance status, and lifestyle choices [112,113]. Unlike traditional regression models [114,115], AI can better handle the complex, non-linear relationships between these factors and survival outcomes, leading to more accurate and individualized predictions that could improve treatment strategies.

### 3.4. Molecular Biomarkers in Laryngeal Squamous Cell Carcinoma

Biomarkers, measurable indicators of biological states, are increasingly vital in understanding LSCC. They can refine diagnosis, predict prognosis, and guide treatment. Research has explored a wide range of molecules as potential biomarkers in LSCC, categorized by their biological roles: long noncoding RNAs (lncRNAs), cell cycle regulators, apoptosis regulators, oncogenes and tumour suppressors, growth factor pathway components, angiogenic factors, structural proteins, sex hormone signalling components, and immunological markers [116]. Table 3 summarize the important biomarkers and their function in laryngeal cell cancer.

**Figure 2:**
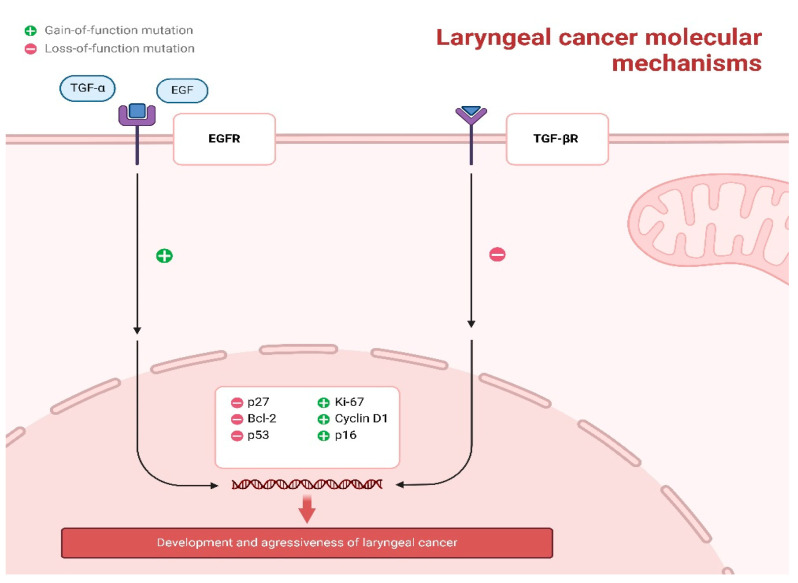
Core Molecular Pathways Driving Laryngeal Squamous Cell Carcinoma Pathogenesis.

Alterations that enhance the function of the epidermal growth factor receptor (EGFR) axis and defects that diminish the function of the transforming growth factor-β receptor (TGF-βR) axis collectively promote unregulated cellular proliferation, reduced apoptosis, and increased cell survival. In the context of EGFR, overexpression or activating mutations result in the receptor being persistently active, thereby continuously stimulating the RAS–RAF–MEK–ERK and PI3K–AKT signaling pathways. This activation leads to the upregulation of pro-proliferative markers such as Cyclin D1 and Ki-67, as well as anti-apoptotic proteins like Bcl-2, while concurrently downregulating cell-cycle inhibitors such as p27. Conversely, the loss of TGF-βR signaling, due to receptor mutations or disruptions in the SMAD pathway, impairs its normal growth-inhibitory functions, resulting in decreased levels of p53 and p27. This loss effectively removes critical regulatory mechanisms that inhibit cell-cycle progression and apoptosis. These opposing molecular events converge within the nucleus, driving DNA replication, promoting survival under stress conditions, and ultimately contributing to the development and aggressiveness of laryngeal cancer (Figure 2).

Biomarkers in laryngeal squamous cell carcinoma (LSCC) encompass a wide range of molecules that modulate key cellular processes and may impact tumor behavior and clinical outcomes.

Long noncoding RNAs (lncRNAs) are RNA molecules that do not code for proteins but regulate gene expression and various cellular functions. Their dysregulation is linked to cancer development, including LSCC, where many lncRNAs are upregulated in tumor tissues. This overexpression often promotes tumor progression, enhances metastasis, reduces radiosensitivity, and worsens overall survival, partly by activating pathways such as Wnt, Sox-2, and TGF-β1 [145,146,147,157]. Owing to their stability in body fluids and ease of detection by non-invasive methods, lncRNAs are considered promising biomarker candidates [158].

The cell cycle, which is frequently disrupted in cancer, offers several potential biomarkers. Ki-67, a nuclear protein expressed during all active phases of the cell cycle but absent in resting cells, serves as a direct marker of proliferation. In LSCC, elevated Ki-67 levels are associated with poorly differentiated tumors, advanced TNM stages, increased recurrence risk, and shorter disease-free survival [133,134,135,136]. However, its relationship with radiotherapy response is complex; while high levels might imply increased radiosensitivity in some studies [159,160], other reports suggest that low Ki-67 can be linked to better local control in early-stage tumors, possibly due to factors like DNA repair capacity and hypoxia [161,162]. Cyclin D1, which drives the G1 to S phase transition by complexing with CDKs 4 and 6, is commonly overexpressed in LSCC and correlates with lymph node metastasis and poorer survival; its overexpression may also occur alongside TP53 mutations [131,139,140]. In contrast, the cyclin-dependent inhibitor p27 is frequently underexpressed in LSCC, with low levels linked to tumor recurrence and poorer outcomes [140,163]. An inverse relationship between cyclin D1 and p27 is evident, with patients displaying cyclin D1 positivity alongside p27 negativity often faring worse [116]. In addition, p16—a CDKI closely related to the HPV 16 status frequently seen in head and neck cancers—is typically downregulated in tumors with high cyclin D1, and its presence tends to indicate better treatment response and survival [164].

Deregulation of apoptotic pathways is another hallmark of LSCC. Bcl-2, an intracellular membrane protein that inhibits apoptosis by modulating cytochrome C release and interacting with pro-apoptotic proteins, shows conflicting associations. While some studies report no correlation between Bcl-2 expression and clinical outcomes [117,118,119,120,121], other investigations have linked its overexpression to lymph node metastasis, advanced stage, poor differentiation, increased recurrence, and radioresistance [165]. A meta-analysis by Silva et al., (2023) even suggests that Bcl-2 overexpression may be associated with poorer lymph node metastasis, overall survival (OS), and disease-free survival (DFS), although these findings should be interpreted with caution because of study variability and potential bias [166].

Tumor suppressor and oncogenes also play crucial roles in LSCC. The tumor suppressor p53, which activates cell cycle arrest, DNA repair, senescence, or apoptosis in response to stress, is mutated in approximately 60–80% of LSCC cases [167,168,169]. These mutations are frequently linked to more aggressive disease features; however, consensus on the prognostic role of p53 remains elusive due to heterogeneous and sometimes conflicting study results [170,171,172,173].

Growth factor signaling contributes further to LSCC progression. Epidermal growth factor receptor (EGFR), a transmembrane receptor tyrosine kinase that binds ligands such as EGF and TGF-α, triggers intracellular pathways leading to increased cell proliferation, angiogenesis, and survival. EGFR overexpression in LSCC is associated with progression to malignancy, a higher risk of metastasis, and reduced survival, particularly when found together with high levels of cyclin D1 and Ki-67 [173,174,175,176,177]. Similarly, alterations in the TGF-β pathway—most notably the loss of TGF-β receptor II expression in lesions progressing to invasive carcinoma—reduce the growth-suppressive effects of TGF-β and promote tumor advancement [169,170,171,172,173,174,175,176,177,178].

Angiogenesis, a process vital for tumor growth and metastasis, is reflected by several markers in LSCC. Vascular endothelial growth factor (VEGF) increases vascular permeability and stimulates endothelial cell proliferation and migration, and its upregulation in LSCC correlates with dysplasia progression, local recurrence, metastasis, and shorter disease-free survival, though some studies find no significant association [126,150,179,180,181,182]. Additional angiogenic markers such as angiogenin and CD105 (endoglin) are also implicated, with their elevated expression correlating with recurrence, advanced disease, and poorer clinical outcomes [126,148,149,150,183,184].

Structural proteins that maintain cell adhesion and tissue integrity are also significant. E-cadherin, a transmembrane glycoprotein essential for cell-cell adhesion, is often downregulated in LSCC, correlating with poorer tumor differentiation, increased risk of nodal metastasis, and advanced tumor stage; reduced expression may also be linked to shorter disease-free survival [125,126,127,185]. The cell surface glycoprotein CD44, a known marker of cancer stem cells, is similarly associated with higher tumor grade and poorer 5-year survival [122,123,124]. Overexpression of focal adhesion kinase (FAK) and cortactin—an actin-binding protein involved in cell motility and invasion—has been linked to aggressive behavior and recurrence in LSCC [126,151,152,153,154].

The role of sex hormone signaling in LSCC remains debated. Estrogen receptors (ERα and ERβ, including variants such as ERα66 and ERα36) display variable expression patterns; while early-stage LSCC tumors may show increased levels compared to normal tissue, advanced stages often exhibit a shift in receptor subtype expression that correlates with a more unfavorable prognosis [155,186,187]. Likewise, progesterone receptor (PR) expression is higher in poorly differentiated LSCC and those with nodal metastasis, whereas androgen receptor (AR) expression is typically reduced in invasive tumors. Elevated prolactin receptor (PRLR) expression has also been noted in LSCC and correlates with poorer survival outcomes [155,156].

Immunological biomarkers reflect the critical role of the host immune response in modulating tumor growth. Tumor-infiltrating lymphocytes (TILs), particularly cytotoxic CD8+ T cells, are associated with improved survival in LSCC—even in cases of tobacco-related disease [188,189,190]. LSCC continues to have a poor prognosis, with a 5-year survival rate of 50 to 60% and suboptimal functional outcomes. The expression of PD-L1 and the tumor microenvironment markers (CD4, CD8, CD68, and CD163) were examined in LSCC using immunohistochemistry. PD-L1 expression demonstrated a statistically significant positive correlation with all the tumor microenvironment cells studied. Higher expressions of CD68 and CD163 were significantly associated with worse clinical outcomes in LSCC patients [191]. To determine which LSCC patients might benefit from immunomodulation therapies, it is crucial to understand the relationship between PD-L1 expression, immune cell distribution, and prognosis. Conversely, the expression of programmed death-ligand 1 (PD-L1) on tumor and immune cells, which aids tumors in evading immune attacks, has been linked to clinical outcomes and may make tumors amenable to immune checkpoint inhibitor therapy [140,192,193,194,195].

Emerging biomarkers in LSCC continue to broaden our understanding of tumor biology. Among these, heat shock proteins (HSPs) have diverse roles: HSP27 and HSP70 are linked to advanced tumor stage, chemoresistance (particularly to cisplatin), and radiotherapy resistance [196,197,198,199,200,201] whereas HSP47 appears to function as a tumor suppressor, with higher expression correlating with better prognosis and longer overall survival [202]. Metallothioneins (MTs), especially the MT1 and MT2 isoforms, are found at higher levels in malignant laryngeal lesions than in benign or dysplastic ones, and genetic variations in MT2A may increase LSCC risk, making them potential early biomarkers [203,204,205,206]. Components of the oxidative stress response, such as nuclear factor erythroid 2-related factor 2 (Nrf2) and heme oxygenase-1 (HO-1), are also implicated; increased nuclear Nrf2 expression correlates with cisplatin resistance, while HO-1 may counteract cisplatin-induced apoptosis, although its prognostic role in LSCC is still under investigation [207,208,209,210,211,212]. Cyclooxygenase-2 (COX-2) is another emerging marker; its elevated expression in LSCC is linked to enhanced angiogenesis, inflammatory signaling, and resistance to radiotherapy, and it is associated with poorer clinical outcomes [213,214,215,216,217,218,219]. Lastly, several microRNAs (miRNAs)—such as Hs_miR-21_5p, Hs_miR-218_3p, and Hs_miR-210_3p, which are expressed exclusively in malignant laryngeal lesions—are implicated in regulating tumor cell migration, invasion, and treatment resistance, further supporting their utility as precise biomarkers [220,221,222,223,224,225,226,227].

### 3.5. Metastatic Patterns of LC

LC metastasis predominantly follows a lymphatic route, with the specific pattern of spread heavily influenced by the primary tumour’s location within the larynx. The supraglottis, possessing a rich lymphatic network and a midline position, exhibits a high likelihood of bilateral lymph node involvement. Lymphatic drainage from the supraglottis flows through the thyrohyoid membrane into the jugular chain, making the jugular lymph nodes the primary recipients of metastases. Specifically, the sub-digastric (level II), mid-jugular (level III), and lower jugular (level IV) nodes are most frequently affected. In contrast, posterior cervical nodes (level V) are rarely involved, and submandibular (level IB) and submental (level IA) nodes are seldom involved. Approximately 55% of patients with supraglottic carcinoma present with clinically involved lymph nodes at diagnosis, with 16% showing bilateral involvement.

Furthermore, up to half of clinically node-negative patients may harbour occult nodal metastases. The risk of lymph node involvement increases with tumour size and grade, reaching approximately 40% for T1 and T2 tumours and 60% for T3 and T4 lesions [228,229,230]. In contrast, the subglottis has a less developed lymphatic network, resulting in a lower incidence of lymph node metastasis (20% to 50%). Subglottic lymphatic channels drain anteriorly through the cricothyroid membrane to the middle and lower jugular or Delphian nodes and posterolaterally to the paratracheal nodes. The true vocal cords are almost devoid of lymphatics, leading to a very low incidence of nodal metastasis in early-stage (T1) glottic cancers, approaching zero. This incidence increases with stage: 2% for T2, 15–20% for T3, and 20–30% for T4 lesions. Occult nodal involvement is found in approximately 16% of clinically node-negative T3 and T4 glottic cancers [229,230]. Glottic cancer spread typically involves extension into the supraglottis or subglottis, leveraging their more prosperous lymphatic supply. Distant metastasis is relatively uncommon in LC, occurring in 10–20% of patients, predominantly those with supraglottic and subglottic primaries, although autopsy studies reveal a higher rate of subclinical metastases. The lung is the most frequent site of distant metastasis, followed by bone (20% of patients with distant disease) and liver (10% clinically). Brain metastases are rare [229,230,231]. Because of the high risk of second primary cancers in these patients, tissue confirmation of suspected metastases is essential. Factors increasing the risk of distant metastasis include lymph node involvement, low neck metastases, advanced stage, and extranodal extension (ENE), with ENE potentially increasing the risk tenfold [19,232].

### 3.6. Recent Trials Further Investigating Biomarkers in LC

Several clinical trials are investigating the role of biomarkers in LSCC treatment and prognosis. One study focusing on locally advanced LSCC treated with radiotherapy found that high pre-treatment expression of the hypoxia marker HIF-1α, along with the presence of regional lymph node metastases, were independent predictors of locoregional recurrence [233]. Another trial examining advanced LC patients primarily treated with surgery found that while high tumour tissue expression of CD4+ and CD8+ T cells initially appeared to improve survival, only higher serum levels of the cytokine IL-8 were a significant negative predictor of disease-specific survival in multivariate analysis [234]. A third study examined inflammatory biomarkers in LSCC patients undergoing definitive radiotherapy, finding that pre-treatment C-reactive protein (CRP) levels were a significant predictor of progression-free survival, while neutrophil-to-lymphocyte ratio (NLR) and ECOG performance status predicted overall survival. Additionally, this study identified that monocyte-to-lymphocyte ratio (MLR), pan-immune inflammatory value (PIV), and CRP levels were significantly higher in patients with lymphatic metastasis [235].

### 3.7. Treatment

The treatment of LC is a multifaceted endeavor, with approaches tailored to the cancer’s stage, location, and the patient’s overall condition. For early-stage (T1-T2, N0, M0) LCs, treatments often aim to preserve the larynx. These can involve laryngeal-sparing surgeries, which selectively remove cancerous tissue, or definitive radiotherapy (RT), employing high-energy beams to eradicate cancer cells [236]. Table 4 summarizes the treatment modalities of LC.

Currently, the standard treatment for locally advanced LC, when aiming for larynx preservation, is concurrent chemoradiation therapy (CRT). This approach combines high-dose cisplatin chemotherapy with radiation therapy, delivered simultaneously. The effectiveness of this concurrent approach was established by the RTOG 91-11 trial, which demonstrated superior larynx preservation rates compared to induction chemotherapy followed by radiation or radiation alone [237]. Intensity-modulated radiation therapy (IMRT) is a widely used technique that allows for highly conformal treatment plans, minimizing radiation dose to sensitive structures like the spinal cord, oesophagus, and salivary glands, thereby reducing toxicities [238].

Beyond the standard CRT approach, several other therapies and considerations are relevant in the treatment of LC. For early-stage disease, definitive radiotherapy alone or laryngeal-sparing surgery may be sufficient [236].

Alternative systemic therapies may be considered for patients who are not candidates for cisplatin. Although cetuximab, an EGFR inhibitor, can enhance radiation’s effects, it has shown inferior overall survival compared to cisplatin in certain head and neck cancers [239]. Thus, for platinum-ineligible cases, carboplatin and fluorouracil, or cetuximab alone, might be considered based on improvements in survival compared to radiation alone in clinical trials.

Immunotherapy, specifically with checkpoint inhibitors like pembrolizumab and nivolumab, is currently approved for recurrent or metastatic head and neck squamous cell carcinoma (HNSCC) [240]. Pembrolizumab and nivolumab received FDA approval in 2016 [240,241]. In the pivotal CheckMate 141 Asian subset, nivolumab conferred a median overall survival of 12.1 months vs. 6.2 months with investigator’s choice therapy, and the estimated 2-year OS rates were 22.7% vs. 0%, respectively; notably, patients who developed any treatment-related adverse events particularly skin-related disorders experienced superior survival, suggesting that early immune-related toxicity may predict benefit [242]. Multiple clinical trials are actively investigating the role of these agents in combination with radiation therapy for locally advanced LC, exploring their potential as alternative radiosensitizers [243,244]. For example, the multinational, Phase III, double-blind, placebo-controlled JAVELIN Head and Neck 100 trial (NCT02952586) is assessing whether adding the PD-L1 inhibitor avelumab to standard cisplatin-based chemoradiotherapy improves progression-free and overall survival compared with chemoradiotherapy plus placebo in patients with high-risk, nonmetastatic, locoregionally advanced HNSCC [245].

Similarly, the Phase III, double-blind, placebo-controlled KEYNOTE-412 trial (NCT03040999) evaluated the addition of pembrolizumab (200 mg q3w) to standard cisplatin-based chemoradiotherapy (70 Gy in 35 fractions) in 804 patients with newly diagnosed, high-risk, unresected, locoregionally advanced HNSCC. Pembrolizumab was given one dose before CRT, two doses during CRT, and up to 14 maintenance doses thereafter. After a median follow-up of 47.7 months, median event-free survival was not reached in the pembrolizumab arm versus 46.6 months in the placebo arm (hazard ratio 0.83 [95% CI 0.68–1.03]; log-rank *p* = 0.043, above the prespecified threshold of *p* ≤ 0.024), and median overall survival was not reached in either arm. Grade ≥3 adverse events occurred in 92% of pembrolizumab-treated versus 88% of placebo-treated patients, with similar safety profiles and no new signals [246]. This trial highlights the challenge of integrating PD-1 inhibitors into definitive CRT for locally advanced HNSCC and underscores the need for biomarker-driven strategies.

Pembrolizumab, a highly selective humanized monoclonal antibody, is designed to disrupt the PD-1 immune checkpoint pathway. It works by binding to the PD-1 receptor on T cells, preventing it from interacting with its ligands, PD-L1 and PD-L2, which are often expressed on tumour cells and other cells in the tumour microenvironment. This blockade releases the “brake” on the immune system, allowing T cells to become activated and attack cancer cells. In the context of recurrent or metastatic head and neck squamous cell carcinoma (HNSCC), including LSCC, the Keynote 040 study investigated the effectiveness of pembrolizumab against standard treatments. In this trial, 247 participants were randomly assigned to receive either pembrolizumab or one of the following standard-of-care therapies: methotrexate (40–60 mg/m^2^), docetaxel (30–40 mg/m^2^), or cetuximab (250 mg/m^2^ weekly after an initial 400 mg/m^2^ loading dose). The median overall survival for patients treated with pembrolizumab was 8.4 months, compared to 6.9 months for those receiving standard therapy. This difference represented a statistically significant improvement in survival for the pembrolizumab group, with a hazard ratio (HR) of 0.80 (95% CI 0.65–0.98; *p* = 0.0161) [247]. The benefit of pembrolizumab was even more pronounced in patients whose tumours had a high proportion of cells expressing PD-L1, as indicated by a tumour proportion score (TPS) of 50% or greater. In this subgroup, the median overall survival was 11.6 months with pembrolizumab versus 6.6 months with standard care, demonstrating a more substantial and statistically significant improvement (HR: 0.53, 95% CI 0.35–0.81; *p* = 0.014) [247].

CTLA-4 inhibitors, such as ipilimumab and tremelimumab, block the CTLA-4 immune checkpoint on T cells preventing its engagement with CD 80/86—and thereby sustain T-cell activation to enhance antitumor immunity. In CheckMate 651 (NCT02741570), 947 patients with previously untreated R/M SCCHN were randomized to first-line nivolumab + ipilimumab versus the EXTREME regimen. Neither the overall population (median OS 13.9 vs. 13.5 months; HR 0.95; *p* = 0.4951) nor the PD-L1 CPS ≥ 20 subgroup (17.6 vs 14.6 months; HR 0.78; *p* = 0.0469) met the primary OS endpoint, although nivolumab + ipilimumab had fewer grade 3–4 treatment-related adverse events (28.2% vs. 70.7%) [248]. In the phase III KESTREL trial (NCT02551159), neither tumor PD-L1 expression (TC ≥ 50%/IC ≥ 25% or TC ≥ 25%) nor a low neutrophil-to-lymphocyte ratio (≤7) enriched for improved outcomes with first-line durvalumab (D) or durvalumab + tremelimumab (D + T) versus EXTREME. However, in patients whose blood tumor mutational burden was ≥16 mut/Mb, D + T achieved a median OS hazard ratio of 0.69 (95% CI 0.39–1.25) and a complete response rate of 8.6% versus 4.3% with EXTREME, suggesting bTMB may help identify those most likely to benefit from checkpoint blockade [249].

In the phase III KESTREL trial (NCT02551159), first-line durvalumab monotherapy or durvalumab plus tremelimumab was compared with EXTREME in recurrent/metastatic HNSCC, and archival tumors or blood were assayed for PD-L1, blood tumor mutational burden (bTMB), and neutrophil-to-lymphocyte ratio (NLR). PD-L1 expression (TC ≥ 50%/IC ≥ 25% or TC ≥ 25%) and NLR ≤ 7 failed to enrich for either overall survival or response rates, whereas in the bTMB ≥ 16 mut/Mb subgroup, durvalumab plus tremelimumab achieved an OS hazard ratio of 0.69 (95% CI 0.39–1.25) versus EXTREME and doubled the complete response rate (8.6% vs. 4.3%), suggesting high bTMB may identify patients most likely to benefit from PD-L1/CTLA-4 blockade.

In the phase III CheckMate 651 trial, first-line nivolumab plus ipilimumab did not improve overall survival compared with the EXTREME regimen in patients with recurrent or metastatic SCCHN, yielding a median OS of 13.9 versus 13.5 months in the intent-to-treat population (HR 0.95, *p* = 0.495) and 17.6 versus 14.6 months in those with PD-L1 CPS ≥ 20 (HR 0.78, *p* = 0.047), despite a substantially lower rate of grade 3–4 treatment-related adverse events (28.2% vs. 70.7%). Among responders, the duration of response was markedly longer with nivolumab/ipilimumab (32.6 vs. 7.0 months), but progression-free survival and response rates were similar between arms.

Proton therapy represents another evolving radiation modality. It offers the potential for a more favourable therapeutic window compared to photons due to the Bragg Peak phenomenon, where the majority of the radiation dose is deposited at a specific depth, minimizing the dose beyond the target. However, access to proton therapy is limited, and prospective, multi-institutional data comparing it to IMRT are still emerging [250]. Stereotactic body radiation therapy (SBRT), which delivers very high doses of radiation in a few fractions, is being investigated for early-stage glottic cancers but is not yet established for laryngeal preservation and should only be used within a clinical trial [251].

While many treatments focus on larynx preservation, total laryngectomy (TL) remains a crucial option, particularly for patients with extensive tumour invasion, significant pre-existing swallowing dysfunction, or those who are not candidates for or have failed organ preservation approaches. Total laryngectomy involves the complete removal of the larynx (voice box), resulting in permanent loss of natural voice production. This procedure necessitates the creation of a permanent tracheostoma, an opening in the neck, for breathing. While a significant intervention, TL can be the most effective treatment for achieving complete tumour removal in certain cases. After a total laryngectomy, patients require relearning for tasks like speaking [252].

In addition, a tracheostomy can secure the airway in patients with significant tumour obstruction, allowing them to undergo CRT instead of requiring a total laryngectomy [253].

Minimally invasive surgical techniques have significantly advanced, offering patients less invasive options with potentially improved functional outcomes. Two prominent techniques are transoral laser microsurgery (TLM) and transoral robotic surgery (TORS).

TLM employs a CO_2_ laser to precisely resect laryngeal tumours through the mouth, avoiding external incisions. Unlike open partial laryngectomy, TLM focuses on removing the tumour with margins while preserving normal structures to optimize function. The European Laryngological Society (ELS) has a classification system for cordectomies, with six types based on resection depth and area [254].

TLM’s primary indication is early-stage vocal fold cancers (Tis, T1, and T2), with consideration for some advanced cases [255]. Limitations include tumours with reduced vocal fold mobility from cricoarytenoid joint fixation/infiltration (though usable with muscle infiltration without joint fixation), cricoid cartilage resection, and the necessity to preserve at least one cricoarytenoid unit [256,257]. TLM has also been applied to supraglottic and hypopharyngeal tumours, demonstrating comparable oncological results and better functional outcomes (less tracheotomy, faster swallowing, shorter hospital stays) than open surgeries [258,259].

TLM is a gold standard for early vocal fold cancers due to high disease-free survival, local/regional control, and larynx preservation rates, sometimes exceeding those of radiotherapy. It offers better postoperative and quality-of-life outcomes than open surgery or radiotherapy and is more cost-effective [260,261,262].

TORS uses the da Vinci system, providing 3D visualization, enhanced instrument manoeuvrability, tremor reduction, and improved movement. TORS received FDA approval in 2009 for specific laryngeal, oropharyngeal, and oral carcinomas and benign tumours. TORS is commonly used for cT1-T2 and some selected cT3-T4a oropharyngeal and supraglottic cancers with adequate visualization and instrument access. TORS supraglottic laryngectomy shows similar oncological results to open procedures, with good functional outcomes (rapid oral diet resumption, low permanent tracheotomy rates) [263,264].

TORS’s role in glottic cancer is less defined. Studies are controversial, with the current robotic instrument length not ideally suited. Research indicates that TORS for glottic cancer has exposure issues, higher costs, increased feeding tube and tracheotomy needs, and more complications than TLM [265,266,267].

### 3.8. Survival

The prognosis for patients with LC is strongly correlated with the disease stage at initial diagnosis and treatment. Other factors that play a role in survival are the patient’s general health and smoking cessation. In the United States, the overall 5-year survival rate for LC is 61% [268]. Survival rates decline significantly with the advancing stage. For diseases confined to the larynx (stages I and II), the 5-year survival rate is 78%. However, regional lymph node metastases (stage III) reduce the 5-year survival to 46%, and distant metastases lower it to 34% [269]. Survival outcomes also vary by laryngeal subsite. Glottic cancer generally has the most favourable prognosis, followed by supraglottic cancer, with subglottic tumours having the least favourable outlook [113]. Specifically, the overall 5-year survival for glottic SCC is 77%, reaching 84% for localized disease (stages I and II) but decreasing to 52% with nodal involvement (stage III) and 45% with distant spread [270]. For supraglottic SCC, the overall 5-year survival is 45%, with rates of 61% for localized disease, 46% for stage III, and 30% for metastatic disease [271,272]. Subglottic SCC has an overall 5-year survival rate of 49%, 59% for stages I and II, 38% for stage III, and 44% for metastatic disease [273,274].

The accurate prediction of prognosis in LC is crucial for tailoring treatment strategies. While the TNM staging system provides a foundation, it does not fully capture the heterogeneity of the disease, particularly regarding the impact of lymph node involvement. Therefore, integrating both clinical factors and biomarkers into comprehensive prognostic models is an area of active investigation. Recent efforts have focused on developing nomograms which is a statistical tools that combine multiple variables to provide individualized risk assessments. For instance, Juan et al., (2024) [275] developed and validated nomograms specifically for early-stage LSCC to predict postoperative recurrence-free survival (RFS) and overall survival (OS). Their models incorporated readily available preoperative blood markers (platelet counts, fibrinogen, platelet-to-lymphocyte ratio, systemic immune-inflammation index, and hemoglobin) alongside clinicopathological characteristics (tumor diameter and differentiation degree). These nomograms demonstrated superior predictive accuracy compared to T staging alone [275].

Similarly, Shi et al., (2017) [276] developed nomograms for LSCC patients undergoing neck dissection, incorporating the lymph node ratio (LNR)—a factor reflecting both the extent of nodal disease and the thoroughness of the dissection—along with a comprehensive set of clinical and pathological factors, including T stage, N stage, tumor size and demographics. Their models, validated both internally and externally, also showed improved predictive performance for both OS and cancer-specific survival (CSS) compared to models without LNR and to traditional TNM staging [276]. These studies highlight that readily available clinical parameters and biomarkers can significantly enhance prognostication in LSCC, there is a need for further investigations into developing new scoring systems. Further research that include novel biomarkers discussed in the review may improve current scoring system and therefore, be used by clinicians to decide the optimal personalized treatment.

Quality-of-life (QoL) outcomes are paramount in the treatment of LC, particularly when comparing organ-preserving approaches versus TL. For early-stage (T1-T2) glottic cancers, both TLM and RT aim for larynx preservation, but their impact on voice and QoL can differ. A meta-analysis by Qasem et al., (2024) [277] found no significant difference between TLM and RT in overall voice-related QoL measures, including the Voice Handicap Index-30 (VHI-30). However, TLM was associated with significantly better outcomes in specific acoustic parameters like jitter and shimmer, suggesting potentially improved voice stability [277]. In more advanced T3 glottic cancers, TLM can still be an option, and Chien et al., (2020) [278] showed that carefully selected T3 glottic SCC patients could achieve satisfactory QoL and larynx preservation rates after CO_2_ TLM. Even after total laryngectomy, where the larynx is removed, voice rehabilitation is crucial for QoL [278]. However, Wulff et al., (2020) [279], in a systematic review, found that individuals who underwent TL generally reported worse health-related quality of life (HRQoL) compared to a male normative reference population, though the reported symptom burden was often mild. The heterogeneity and generally low quality of existing studies highlight a need for more rigorous research in this area [279]. These studies, overall, emphasize a need for individualized assessment when choosing a treatment and obtaining a high level of QoL. Table 5 shows a summary of survival data provided within this section.

## 4. Conclusions

LC, a complex and heterogeneous disease, presents significant diagnosis, treatment, and prognostication challenges. This review has highlighted the crucial role of accurate staging in guiding treatment decisions, incorporating both clinical and pathological findings. The distinct histological variants of LSCC, each with unique characteristics and behaviors, necessitate careful pathological evaluation. The expanding knowledge of biomarkers offers the potential for improved risk stratification and personalized treatment approaches, although further validation is needed for many of these markers. While treatment strategies have evolved, with a growing emphasis on organ preservation, the advanced-stage disease continues to pose a significant challenge.

Recognizing that no single specialist can address every nuance of LC care, it is essential to integrate multidisciplinary team (MDT) decision-making throughout the patient journey. Regular MDT reviews, which bring together head and neck surgeons, radiation and medical oncologists, radiologists, pathologists, and allied health professionals, ensure that emerging biomarkers and novel therapies are interpreted within a comprehensive clinical context.

Future research in LC must address several critical gaps to advance the field. A primary focus should be the rigorous validation of promising biomarkers, moving beyond exploratory studies to establish their clinical utility in diverse patient populations and treatment settings. This includes standardizing assays and determining optimal cut-off values for clinical decision-making. Simultaneously, the development of novel targeted therapies is crucial, particularly those addressing specific molecular alterations driving tumor growth and resistance. Emphasis should also be on personalized medicine. This means creating strategies that are individualized and focus on the combination of specific tumoral factors and host factors. This personalized approach necessitates integrating comprehensive genomic and proteomic profiling with detailed clinical data to tailor treatment selection and predict response to therapies. Finally, optimizing treatment strategies, including refining radiation techniques, exploring immunotherapy combinations, and developing strategies to overcome resistance, is essential to improve patient outcomes and quality of life.

## Figures and Tables

**Table 1:** Histopathological and Prognostic Features of Laryngeal SCC Variants.

Variant	Gross Appearance	Microscopic Features & Keratinization	Immunohistochemistry/Special Studies	Prognosis
Conventional SCC	Variable appearance: may be ulcerated or smooth; endophytic, exophytic, or polypoid; colour from red to tan-white; firm texture.	Invasive nests of squamous cells with variable differentiation; abnormal keratinization (prominent in well-differentiated types); desmoplastic reaction; possible perineural/vascular invasion.	In poorly differentiated cases: CK5/6, p63, p40, and EMA positivity help confirm epithelial origin.	Stage-dependent survival rates (e.g., Glottic: 80–85%, Supraglottic: 65–75%, Subglottic: ~40%).
Verrucous Carcinoma	Warty, broad-based, and fungating mass; firm-to-hard texture; generally tan or white.	Well-demarcated pushing border with blunt, club-shaped rete ridges; minimal atypia and rare mitoses; abundant keratin forming “church-spire” structures.	Lacks evidence of transcriptionally active HPV.	Excellent outlook with 5-year survival rates of approximately 85–95% (stage is key).
Papillary/Exophytic SCC	Polypoid and bulky, projecting outward with papillary or fungiform features; texture may vary from soft to firm.	Dominantly exophytic/papillary growth pattern with malignant features; surface keratinization; possible koilocytic atypia.	Diagnosis is usually made on morphology; additional studies are not typically required.	Generally favorable outcome with roughly 85% 5-year survival, better than conventional SCC.
Spindle Cell (Sarcomatoid) SCC	Often presents as a polyp-like mass with areas of surface ulceration; firm, fibrous texture.	Biphasic pattern showing both conventional SCC and atypical spindle cell components; high cell density, pleomorphism, and increased mitoses; keratinization is often focal.	Approximately 70% of cases show AE1/AE3, EMA, p63, and/or p40 positivity; frequent p53 overexpression.	Generally excellent, sometimes even better than conventional SCC.
Basaloid SCC	Firm to hard mass, frequently with central necrosis.	Deep invasive lobules of basaloid cells with a high nuclear-to-cytoplasmic ratio; palisading at the periphery; abrupt squamous differentiation with focal keratinization; comedonecrosis is common; may show hyaline stroma.	Consistent epithelial marker expression (e.g., pan-cytokeratin, p63, p40) and p53 overexpression; typically negative for neuroendocrine markers.	Tends to have a worse overall prognosis compared to conventional SCC.
Adenosquamous Carcinoma	Typically presents as a submucosal, indurated mass.	Infiltrative growth with two distinct components: a conventional squamous carcinoma element and an adenocarcinoma (glandular) component; abrupt or focal keratinization (keratin pearls may be seen).	Glandular areas are positive for mucin, supporting a dual differentiation profile.	When matched for stage, outcomes are similar to conventional SCC, though the lesion may behave more aggressively.

**Table 2:** Diagnostic Modalities in Laryngeal Cancer: Techniques, Strengths, and Limitations.

Method	Description	Advantages	Limitations
Clinical Examination	Patient history, physical exam (neck palpation, etc.)	The essential first step is to identify symptoms and risk factors.	Cannot visualize the larynx directly; subjective.
Laryngoscopy	Indirect, flexible fiberoptic, video stroboscopy	Direct visualization of the larynx; videostroboscopy assesses vocal fold vibration.	It can be uncomfortable and may require local anaesthesia and inter-observer variability.
Biopsy	A tissue sample from the primary tumour or lymph nodes (fine-needle aspiration)	The gold standard for definitive diagnosis allows for histological analysis.	Invasive; potential for complications (bleeding, infection); sampling error.
Imaging	CT, MRI, PET/CT	CT: assesses bone involvement; MRI: superior for soft tissue and cartilage; PET/CT: detects recurrences and metastases.	CT: radiation exposure; MRI: cost, claustrophobia; PET/CT: cost, availability, radiation exposure.
Narrow-band Imaging (NBI)	Uses specific wavelengths of light to enhance visualization of mucosal changes	High sensitivity and specificity for identifying LC and precursor lesions.	It requires specialized equipment; it may not detect deep invasion.
Liquid Biopsy	Analysis of biomarkers in blood/saliva (CTCs, ctDNA, exosomes, microbiome)	Minimally invasive; potential for early detection, prognosis, and monitoring treatment response.	Biomarker validation is ongoing; standardization is needed; it may not replace tissue biopsy.
AI-Assisted Diagnosis	Machine learning/deep learning applied to imaging (histology, endoscopy)	Potential to improve diagnostic accuracy and efficiency; personalized risk assessment.	It requires large datasets for training, the “black box” nature of some algorithms, and ethical considerations.

**Table 3:** Molecular Biomarkers in Laryngeal SCC: Biological Roles and Clinical Implications.

Biomarker (Symbol)	Primary Function	LC-Specific Application	Clinical Impact
BCL2	Suppresses apoptosis	IHC to identify tumors with high anti-apoptotic tone	Overexpression correlates with radio-/chemo-resistance and poorer OS and DFS [117,118,119,120,121]
CD44	Regulates cell–cell/ECM interactions; CSC marker	CSC enrichment assays; marker for minimal residual disease	High CD44^+^ fraction associates with increased recurrence and metastasis [122,123,124]
E-cadherin (CDH1)	Mediates calcium-dependent cell–cell adhesion	IHC loss as indicator of EMT	Reduced expression predicts invasion, nodal spread, and worse prognosis [125,126,127,128]
p16 (CDKN2A)	Inhibits CDK4/6; surrogate of HPV-driven oncogenesis	p16 IHC to stratify HPV^+^ vs. HPV^−^ tumors	p16 positivity correlates with better OS in HPV-associated LC [129]
p27 (CDKN1B)	Restrains cyclin-CDK activity	IHC gauge of intact growth-inhibitory signaling	Low p27 levels associate with aggressive phenotype and reduced DFS [130]
Cyclin D1 (CCND1)	Drives G_1_–S cell-cycle transition	FISH/IHC for gene amplification and overexpression	Overexpression predicts lymph node metastasis and shorter DFS [131]
EGFR	RTK regulating proliferation, survival, angiogenesis	IHC/FISH to select patients for EGFR-targeted therapies (cetuximab, TKIs)	High EGFR expression correlates with poor prognosis; targetable with cetuximab [132]
Ki-67 (MKI67)	Marks actively cycling cells	Ki-67 index quantification in biopsy specimens	Elevated Ki-67 (>30–40%) predicts high tumor grade and reduced survival [133,134,135,136]
PCNA	Cofactor for DNA polymerase δ during replication	IHC metric of S-phase fraction and proliferative activity	High PCNA levels associate with rapid growth and adverse outcome [137]
SPP1 (Osteopontin)	Adhesion molecule and cytokine	Serum/plasma ELISA and tissue IHC	Elevated SPP1 correlates with metastasis, chemoresistance, and shorter OS [138]
p53 (TP53)	Orchestrates DNA-damage response, apoptosis, senescence	Mutational analysis by NGS/IHC for risk stratification	TP53 mutation denotes higher recurrence risk and poor prognosis [131,139,140]
VEGFA	Stimulates angiogenesis and vascular permeability [141]	IHC/ELISA to assess angiogenic index	High VEGFA expression correlates with microvessel density and reduced OS [142,143,144]
TGF-βR	Mediates TGF-β growth-inhibitory signaling	IHC/genomic profiling for receptor or SMAD pathway defects	Loss correlates with invasion, EMT, and poor survival [145,146,147]
Endoglin (CD105)	Accessory TGF-β receptor controlling angiogenesis	IHC microvessel density marker	High CD105^+^ microvessel count predicts aggressive behavior and reduced OS [126,148,149,150]
FAK (PTK2)	Integrates integrin and growth-factor signals to regulate motility	IHC and activity assays; under investigation as therapeutic target	Overexpression linked to metastasis, diminished DFS, and poorer OS [151,152,153,154]
Hormone receptors (ER, PR, AR, PRLR)	Ligand-activated TFs mediating hormone-driven growth and differentiation	IHC detection to explore endocrine manipulation	AR^+^ tumors may be less aggressive; PRLR elevation correlates with poorer survival [155,156]

CSC, cancer stem cell; ECM, extracellular matrix; IHC, immunohistochemistry; FISH, fluorescence in situ hybridization; RTK, receptor tyrosine kinase; TKIs, tyrosine kinase inhibitors; NGS, next-generation sequencing; DFS, disease-free survival; OS, overall survival; ELISA, enzyme-linked immunosorbent assay.

**Table 4:** Treatment Modalities for LC.

Treatment Modality	Description	Indications	Advantages	Disadvantages
Surgery				
Laryngeal Sparing Surgery	Selective removal of cancerous tissue.	Early-stage (T1, T2, N0, M0)	It preserves the larynx and potentially improves voice and swallowing function.	It may not be suitable for advanced disease due to the risk of recurrence.
Transoral Laser Microsurgery (TLM)	CO_2_ laser resection through the mouth.	Early-stage vocal fold cancers (Tis, T1, T2); selected advanced cases.	Minimally invasive; good oncological outcomes; better postoperative and QoL outcomes than open surgery or radiotherapy; cost-effective.	Limited by tumour extent and location; requires specialized equipment and expertise.
Transoral Robotic Surgery (TORS)	Robotic-assisted surgery through the mouth.	Selected supraglottic cancers (cT1,T2, some cT3,T4a); oropharyngeal cancers.	Minimally invasive; improved visualization and manoeuvrability.	Role in glottic cancer less defined; potential for exposure issues, higher costs, and complications compared to TLM.
Total Laryngectomy (TL)	Complete removal of the larynx.	Extensive tumour invasion; significant pre-existing swallowing dysfunction; failed organ preservation.	Definitive tumour removal.	Permanent loss of natural voice requires tracheostomy, which has a significant impact on QoL.
Radiation Therapy (RT)				
Definitive RT	High-energy beams to eradicate cancer cells.	Early-stage disease; alternative to surgery.	Preserves larynx.	Potential for side effects (mucositis, xerostomia, dysphagia); risk of recurrence.
Intensity-Modulated RT (IMRT)	Conformal radiation delivery, minimizing dose to surrounding tissues.	Locally advanced disease (often combined with chemotherapy).	Reduced toxicities compared to conventional RT.	It requires specialized equipment and expertise; there is potential for late effects.
Proton Therapy	Radiation using protons, with the potential for reduced dose to surrounding tissues.	Investigational; potential for locally advanced disease.	It may reduce side effects compared to IMRT.	Limited availability, cost, and lack of robust comparative data.
Stereotactic Body RT (SBRT)	High doses of radiation in a few fractions.	Investigational; potential for early-stage glottic cancers (clinical trial only).	Short treatment course.	Not established for laryngeal preservation; potential for severe toxicities.
Chemotherapy				
Concurrent Chemoradiotherapy (CRT)	Cisplatin chemotherapy combined with radiation therapy.	Locally advanced disease (standard of care for larynx preservation).	Superior larynx preservation rates compared to induction chemotherapy or radiation alone.	Significant toxicities (mucositis, nephrotoxicity, myelosuppression).
Alternative Systemic Therapies	Carboplatin/fluorouracil, cetuximab (for platinum-ineligible patients).	Patients not candidates for cisplatin.	It may be better tolerated than cisplatin.	Cetuximab may have inferior overall survival compared to cisplatin in certain cases.
Immunotherapy	Pembrolizumab, Nivolumab (checkpoint inhibitors)	Investigational; potential for locally advanced disease. approved for recurrent or metastatic disease.	It may reduce side effects compared to IMRT.	Limited availability; cost; lack of robust comparative data.
Tracheostomy	Creating a hole in the neck to breathe	In cases of significant tumor obstruction	Secures the airway in patients and allows patients to get CRT.	Patient will have to relearn to speak

**Table 5:** Survival Rates and Prognostic Factors in LC.

Factor	Subcategory/Details	Impact on Survival
Stage (TNM)	I & II (localized)	Showed the best prognosis. 5-year survival: 78% [269].
	III (regional lymph node involvement)	Showed an intermediate prognosis. 5-year survival: 46% [269].
	IV (distant metastases)	Showed the poorest prognosis. 5-year survival: 34% [269].
Laryngeal Subsite	Glottic	Generally best prognosis; overall 5-year survival: 77%; localized: 84%; nodal involvement: 52%; distant spread: 45% [270].
	Supraglottic	Overall 5-year survival: 45%; localized: 61%; stage III: 46%; metastatic: 30% [270].
	Subglottic	Worst prognosis; overall 5-year survival: 49%; stages I & II: 59%; stage III: 38%; metastatic: 44% [270].
Lymph Node Status	N0 (no nodal involvement)	Better prognosis.
	N+ (nodal involvement)	Significantly worse prognosis; higher risk of recurrence and distant metastasis.
	Extranodal Extension (ENE)	Significantly increases risk of distant metastasis (potentially tenfold).
Histological Grade	Well-differentiated	Generally better prognosis.
	Poorly differentiated	Generally worse prognosis; higher risk of recurrence and metastasis.
Patient Factors	Age, general health, performance status, comorbidities, smoking	Poorer general health, older age, and continued smoking associated with worse outcomes.
Biomarkers	See Table 3 (multiple biomarkers with varying prognostic value)	Can provide additional prognostic information beyond TNM staging; potential for personalized risk assessment.
Prognostic Models	Nomograms incorporating clinical factors and biomarkers	Can improve prognostication in LSCC.
QoL	TLM, RT, and TL	Overall, voice-related QoL measures are not significantly different between TLM and RT, TLM patients demonstrated better outcomes in specific acoustic measures. Carefully selected T3 glottic SCC patients achieve satisfactory QoL and larynx preservation rates after CO_2_ TLM. Individuals who underwent TL reported worse HRQoL compared to a male normative reference population.
